# Supplementary material for: The causal role of circulating inflammatory markers in osteoporosis: a bidirectional Mendelian randomized study
Source: Front Immunol. 2024 Jul 18;15:1412298. doi: 10.3389/fimmu.2024.1412298 (PMC11291241; doi:10.3389/fimmu.2024.1412298)
Supplement: Supplementary file 1 [file DataSheet_1.zip › supplementary material/CIM-OSTEOPOROSIS /GCST90274809.csv_forest.pdf]

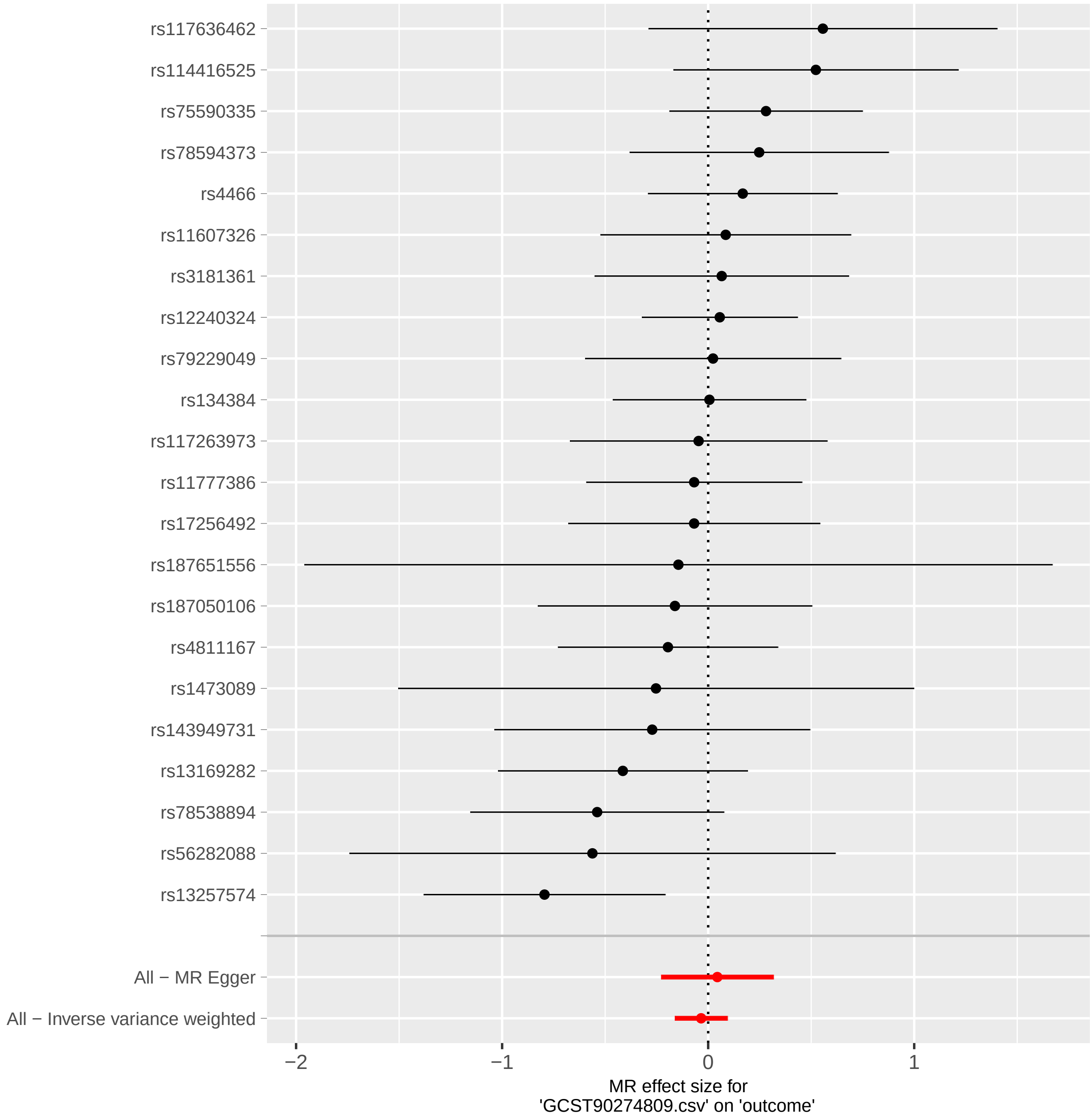
rs117636462
rs114416525
rs75590335
rs78594373
rs4466
rs11607326
rs3181361
rs12240324
rs79229049
rs134384
rs117263973
rs11777386
rs17256492
rs187651556
rs187050106
rs4811167
rs1473089
rs143949731
rs13169282
rs78538894
rs56282088
rs13257574
All − MR Egger
All − Inverse variance weighted
−2
−1
0
1
MR effect size for
'GCST90274809.csv' on 'outcome'
